# Co-alteration of Myc and RTK-RAS pathways defines a liver-metastatic propensity and immune-cold subgroup of pancreatic adenocarcinoma

**DOI:** 10.1016/j.gendis.2023.05.006

**Published:** 2023-06-29

**Authors:** Yuyuan Zhang, Ziyang Zu, Hui Xu, Siyuan Weng, Yuqing Ren, Quan Cheng, Peng Luo, Jian Zhang, Zaoqu Liu, Xinwei Han

**Affiliations:** a Department of Interventional Radiology, The First Affiliated Hospital of Zhengzhou University, Zhengzhou, Henan 450052, China; b Interventional Institute of Zhengzhou University, Zhengzhou, Henan 450052, China; c Interventional Treatment and Clinical Research Center of Henan Province, Zhengzhou, Henan 450052, China; d Department of Hepatobiliary and Pancreatic Surgery, The First Affiliated Hospital of Zhengzhou University, Zhengzhou, Henan 450052, China; e Department of Respiratory and Critical Care Medicine, The First Affiliated Hospital of Zhengzhou University, Zhengzhou, Henan 450052, China; f Department of Neurosurgery, Xiangya Hospital, Central South University, Changsha, Hunan 410008, China; g Department of Oncology, Zhujiang Hospital, Southern Medical University, Guangzhou, Guangdong 510280, China

Co-altered pathways refer to the phenomenon where multiple biological pathways exhibit aberrant changes simultaneously within the same tumor sample. This phenomenon can facilitate a better understanding of the mechanism and evolution of tumors and serve as a biological marker for diagnosing, classifying, and treating tumors.^1^ However, the nature of alteration occurrence and the impact on pancreatic adenocarcinoma (PAAD) remain elusive. The SELECT algorithm was originally designed to systematically assess the evolutionary dependencies and their impact between altered genes in cancer for anticipating drug resistance and proposing alternative strategies. Here, to better characterize the etiology of PAAD and develop an improved risk assessment strategy,^2^ by utilizing SELECT, we identified a co-altered pathway subgroup of PAAD that demonstrated an elevated risk for unfavorable prognosis, a propensity for liver metastasis, and an immunologically cold microenvironment.

A total of 2480 PAAD samples from three independent cohorts with genomic and/or transcriptomic data were enrolled (Table S1). Firstly, the SELECT algorithm was conducted to interrogate the biological evolutionary events occurring in ten canonical pathways of PAAD (Table S2; see supplementary Materials and Methods). Co-occurring alterations were identified in 23 pairs, while mutually exclusive alterations were absent (Fig. 1A and Table S3). Notably, only alterations in Myc and RTK-RAS pathways showed statistical significance and cumulative effect for prognostic stratification (Fig. 1A; Fig. S1A–C and Table S3). To concentrate on the high-risk population within PAAD, patients have been further divided into the Double-Altered (DA) and non-Double-Altered (non-DA, Single-Altered/Double-Unaltered) subgroups. All three cohorts confirmed that *KRAS* mutation, *TP53* mutation, and *MYC* amplification were the dominant genome alterations in maintaining DA phenotype (Fig. S1D–F). Figure 1B–D showed that the DA subgroup remained a significantly adverse prognosis in all cohorts. In addition, multivariate Cox regression confirmed that the DA subgroup could be regarded as an independent prognostic indicator for PAAD when adjusted for available clinical traits (Fig. 1E–G). Recurrence of PAAD is common, with frequent metastases to the liver and peritoneum, and median survival for metastatic PAAD is only 3–6 months.^3^ Using the information about metastasis in the MSK-MET cohort, the incidences of 20 metastatic sites between DA and non-DA were compared (Table S4). We observed a significant increase in the proportion of liver metastasis in DA (Fig. 1H). Expectedly, TCGA-PAAD patients with liver metastasis had adverse prognoses (Fig. 1I). To further elucidate the prognostic significance of liver metastasis on DA, we performed a stratified survival analysis (Fig. 1J). Figure 1I and J suggested that liver metastasis had a more negative impact on the outcome of DA compared to non-DA. Taken together, the tendency to liver metastasis may be a significant clinical feature of Myc/RTK-RAS pathway co-altered PAAD and is detrimental to its patients' survival.

**Figure 1:**
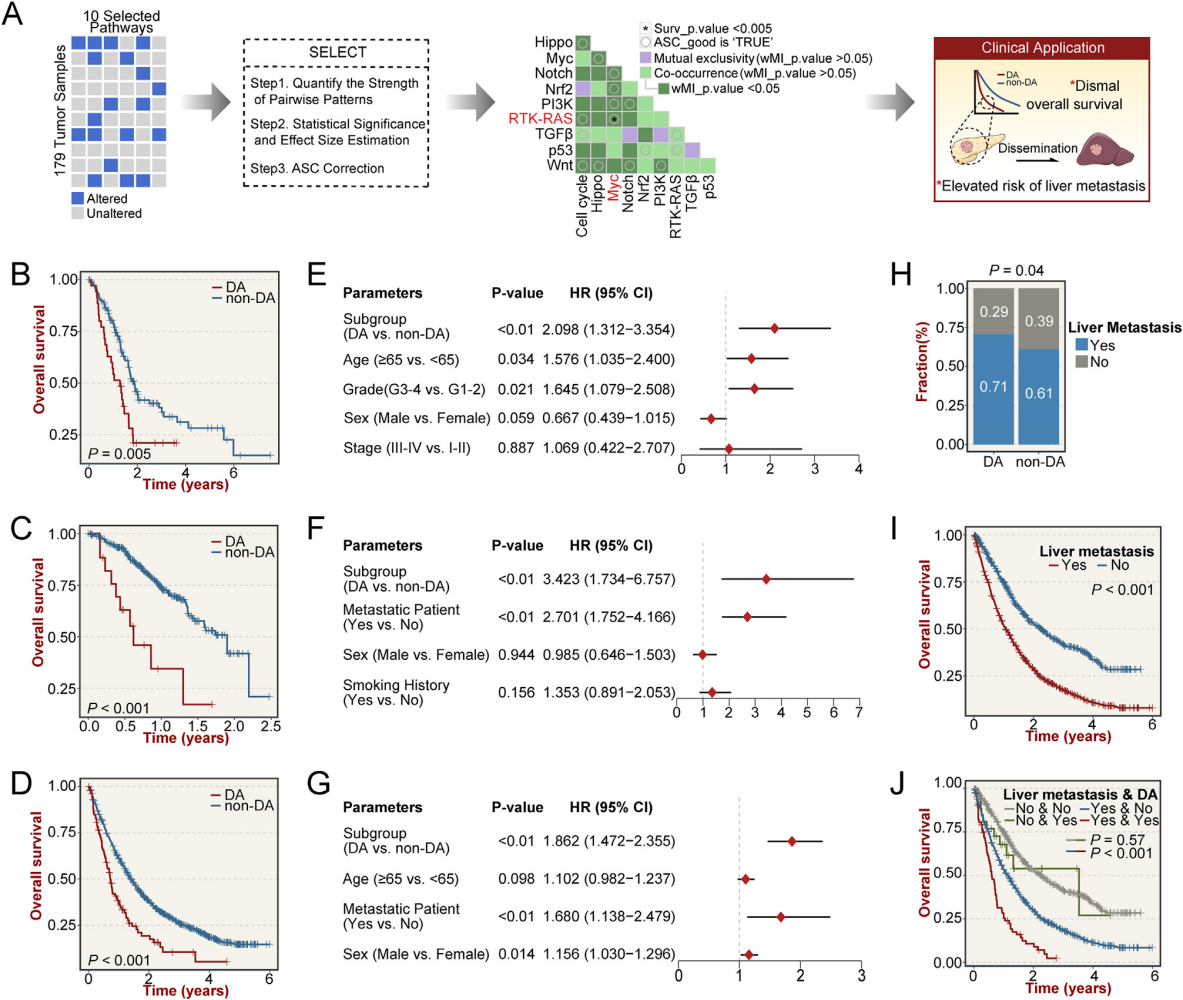
Identification of a pancreatic adenocarcinoma subgroup with co-alteration of Myc and RTK-RAS pathways using the SELECT algorithm. **(A)** Based on the SELECT algorithm, the identification of mutual exclusivity and co-occurrence for oncogenic pathways in the TCGA-PAAD cohort. **(B–D)** Kaplan–Meier survival analyses between the DA and non-DA subgroups in the TCGA-PAAD (B), MSK-IMPACT (C), and MSK-MET (D) cohorts. **(E–G)** Multivariate Cox regression analyses of the DA subgroup in the TCGA-PAAD (E), MSK-IMPACT (F), and MSK-MET (G) cohorts. **(H)** Comparison of liver metastasis rates in the DA and non-DA subgroups in the MSK-MET cohort. **(I)** Kaplan–Meier survival analysis of TCGA-PAAD patients with or without liver metastasis in the MSK-MET cohort. **(J)** Kaplan–Meier survival analyses of the DA and non-DA subgroups with or without liver metastasis in the MSK-MET cohort. ASC, average sum correction; DA, double-altered; non-DA, non-double-altered; HR, hazard ratio; 95% CI, 95% confidence interval.

Given the laborious and costly nature of identifying clinically actionable alterations in the member genes of the Myc and RTK-RAS pathways, we intended to develop a classifier that could accurately identify PAAD with greater clinical relevance. Initially, WGCNA was performed to search for co-expressed genes associated with DA. The optimal β value of 9 was considered as the soft threshold (scale-free *R*^2^ > 0.85, Fig. S2A). Correlations between modules were visualized on an eigengene adjacency heatmap (Fig. S2B). We simplified the network by merging modules with a similarity greater than 0.75 (Fig. S2C). Eventually, 11 modules were identified, of which the blue and purple modules were highly relevant to the subgroups (Fig. S2D). Subsequently, areas under the ROC curves (AUCs) of each gene from the blue and purple modules were calculated. Genes with AUC > 0.7 were retained for exploiting the LASSO regression (Table S5). Model development and validation were executed in training and testing sets, respectively. Ultimately, we extracted 14 genes with optimal lambda and constructed a DA predictor (DApred) (Fig. S2E and Table S6). Due to the absence of appropriate cohorts, the TCGA-PAAD cohort was randomly divided into training (70%) and testing (30%) sets. The detailed AUCs of training (0.885) and testing sets (0.880) were displayed in Figure S2F and G. Our model evaluation indicated that DApred processed excellent prediction efficacy for DA diagnosis.

Then, we investigated phenotype-specific molecular alterations in DA. Firstly, the mutational landscape of the 20 frequently mutated genes (FMGs) in DA and non-DA was delineated. Most FMGs exhibited a higher mutation frequency in DA, especially *KRAS*, *PAADDH15*, and *RYR1* (Fig. S3A). Similarly, the top 15 broad-level copy number variations (CNVs) generally occurred more frequently in DA. Specifically, copy number gains on 8q24.21 (*MYC* position) and 8q24.3 as well as copy number losses on 17p12, 1p36.11, and 8p23.2 represented DA-specific CNVs (Fig. S3B). Afterward, we measured the overall genomic alterations between DA and non-DA in terms of bases, segments, and chromosome arms. In addition to arm gain, CNVs across the genome and chromosomal arm were significantly increased in DA (Fig. S3C, D). Furthermore, the DA subgroup had a significantly higher tumor mutational burden, which further emphasizes the high genomic instability of DA (Fig. S3E).

To investigate the latent biological mechanism underlying the DA subgroup, Gene Ontology (GO) and Kyoto Encyclopedia of Genes and Genomes (KEGG) analyses were performed. In terms of GO analysis, the DA subgroup showed features related to proliferation–relevant processes, such as sister chromatid segregation and ribosome biogenesis, while the declined terms were all associated with immune response (Fig. S4A). Consistently, KEGG analysis revealed up-regulation of proliferation-related pathways like cell cycle, while several immune microenvironment-related pathways, including TGF-β, NOD-like receptor, and FCεRI signaling pathways, were significantly down-regulated in DA (Fig. S4B). Moreover, a total of 30 pathways related to proliferation, metastasis, cell stemness, signaling pathway, cell death, as well as immunity were retrieved to further compare potential tumor behaviors in distinct subgroups. It was observed that DA was characterized by activation of proliferation and metastasis state (Fig. S4C). In contrast, predominant immune-relevant terms, including activation of immune response and complement, were inactive in DA (Fig. S4C). These results suggested that the DA subgroup might elicit diverse biological functions, including enhanced proliferation, metastatic proclivity, and the down-regulation of immune-mediated pathways.

As expected, the DA subgroup exhibited lower infiltration of multiple immune cells, including activated B cells, activated CD8^+^ T cells, effector memory CD8^+^ T cells, and T follicular helper cells (Fig. S5A). Additionally, the DA subgroup also exhibited predominantly lower expression levels of immune checkpoints, such as *CTLA4*, *PD-1*, and *PD-L2* (Fig. S5B). Overall, the DA subgroup is likely to be an “immune cold” subtype. Several critical indicators for evaluating tumor antigenicity and genetic instability provided by Thorsson et al indicated that the DA subgroup had higher homologous recombination defects, nonsilent mutation rate, and silent mutation rate^4^ (Fig. S5C). Furthermore, we matched DA/non-DA with six recently published immune subtypes to explore their potential connections.^4^ It was observed that the DA subgroup probably consisted of more C1 and C2 and fewer C3 and C6 components (Fig. S5D), which might reflect the stronger proliferative capacity and lower lymphocyte infiltration of DA.^4^^,^^5^ In conclusion, the immune features of DA were endowed with immune-cold and high tumor antigenicity.

Overall, our study has demonstrated that the concurrent alteration of Myc and RTK-RAS pathways in PAAD indicated unfavorable prognosis, high liver-metastatic propensity, and immune-cold subgroup. These findings are expected to improve clinical management and targeted therapeutic efficacy for PAAD patients.

## Ethics declaration

We have obtained consent to publish this paper from all the participants of this study.

## Author contributions

Zaoqu Liu, Xinwen Han, and Yuyuan Zhang provided direction and guidance throughout the preparation of this manuscript. Ziyang Zu, Yuyuan Zhang, Hui Xu, and Zaoqu Liu wrote and edited the manuscript. Yuyuan Zhang reviewed and made significant revisions to the manuscript. Hui Xu, Ziyang Zu, Yuyuan Zhang, Siyuan Weng, Jian Zhang, Peng Luo, Quan Cheng, and Yuqing Ren collected and prepared the related papers. All authors read and approved the final manuscript.

## Conflict of interests

The authors declare that they have no competing interests.

## Funding

This study was supported by the Major Science and Technology projects of Henan Province, China (No. 221100310100).
