# Supplementary material for: Co-alteration of Myc and RTK-RAS pathways defines a liver-metastatic propensity and immune-cold subgroup of pancreatic adenocarcinoma
Source: Genes Dis. 2023 Jun 29;11(3):100993. doi: 10.1016/j.gendis.2023.05.006 (PMC10806262; doi:10.1016/j.gendis.2023.05.006)
Supplement: Multimedia component 1 [file mmc1.docx]

# Figures and Figure legends


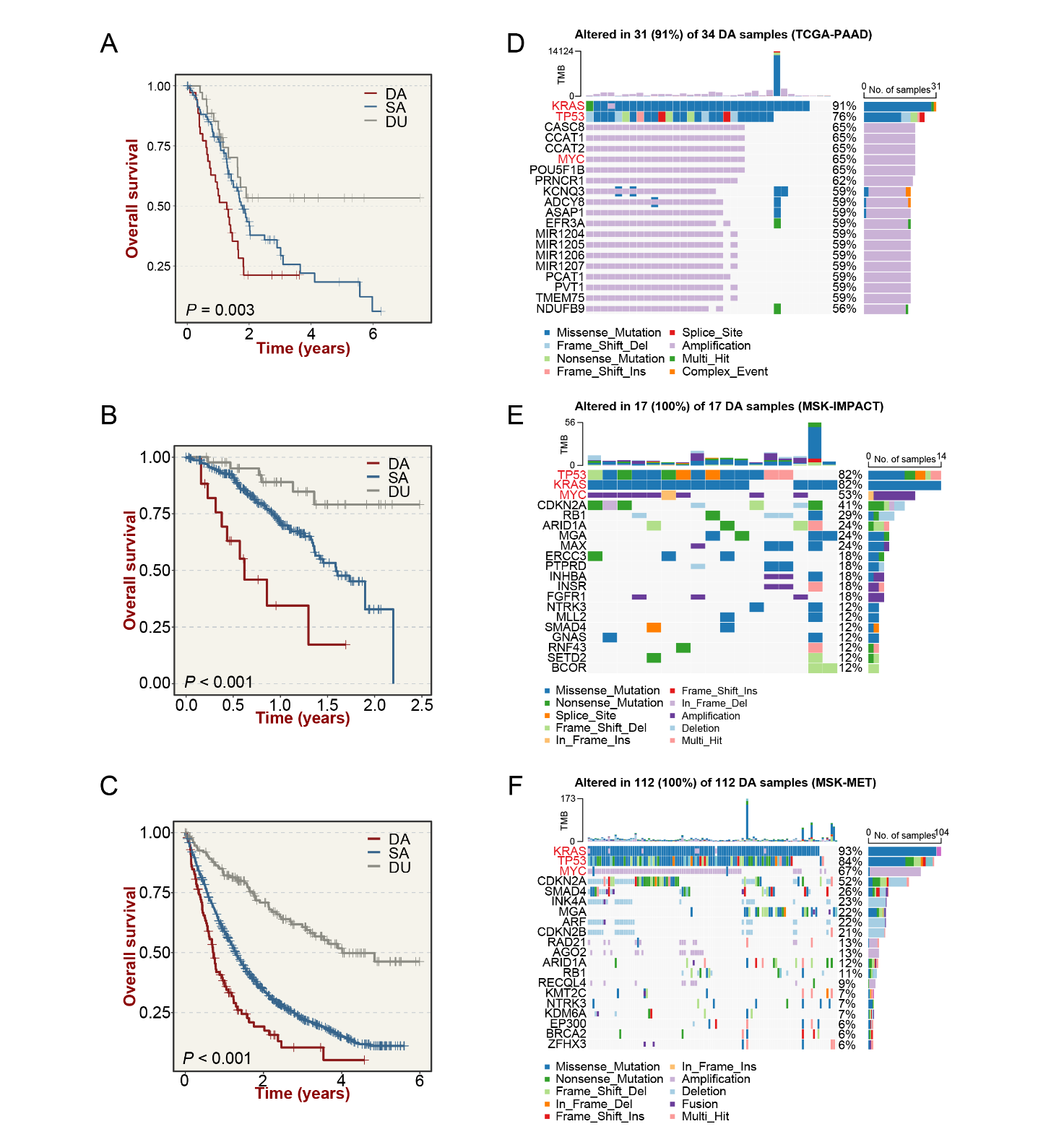


**Figure S1** Kaplan-Meier survival analyses of the DA, SA, DU subgroups and the genomic landscapes of DA in three independent cohorts. **(A-C).** Kaplan-Meier curves of DU, SA, and DA in TCGA-PAAD, MSK-IMPACT, and MSK-MET cohorts. **(D-F).** The genomic alterations of DA in TCGA-PAAD, MSK-IMPACT, and MSK-MET cohorts. DA, Double-Altered; SA, Single-Altered; DU, Double-Unaltered.


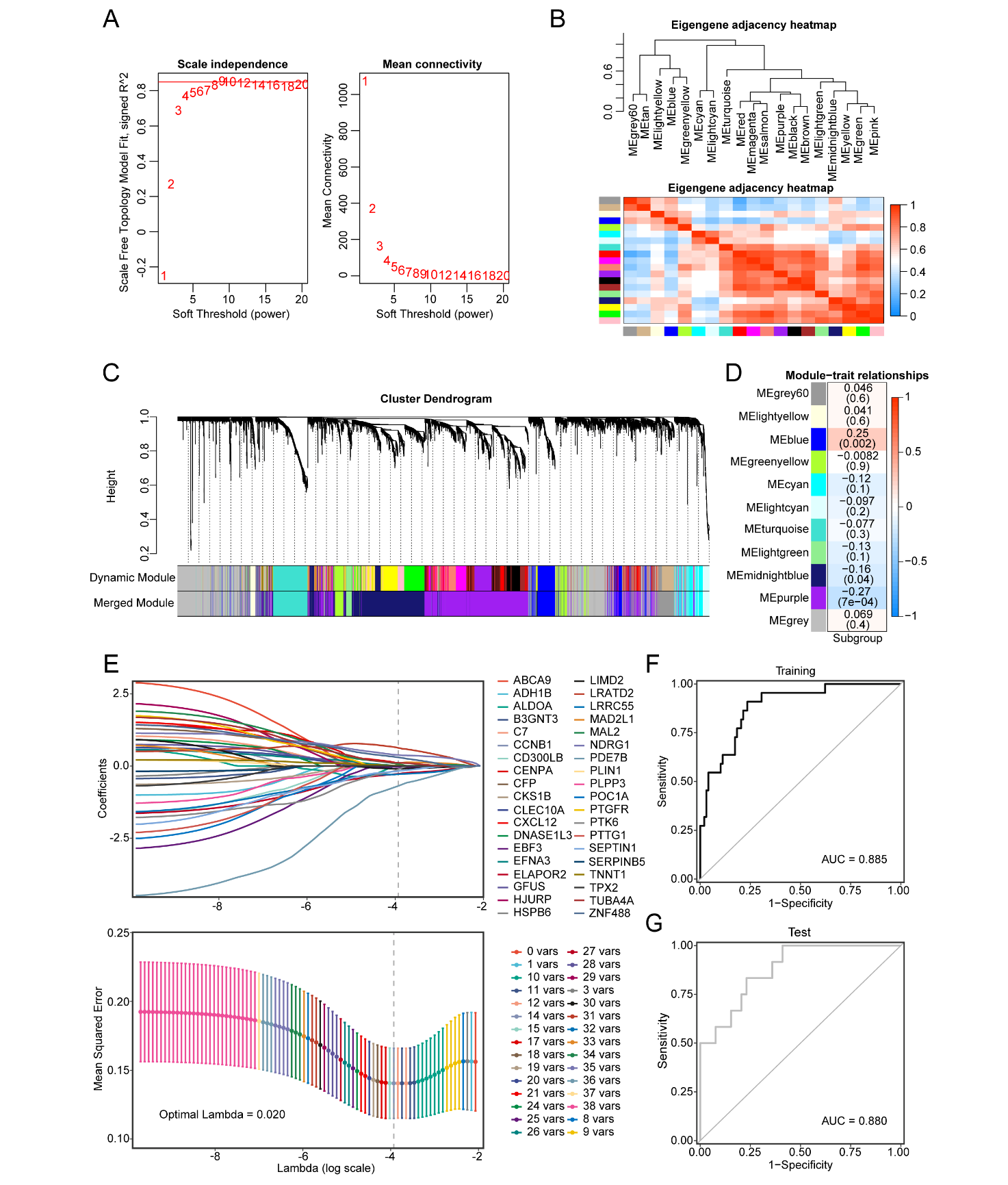


**Figure S2** Modules relevant to the two subgroups and LASSO regression algorithms. **(A).** Scale-free topological indices at various soft-thresholding powers; **(B).** Heatmap of the eigengene adjacency. **(C).** Gene clustering diagram based on hierarchical clustering under optimal soft-thresholding power. **(D).** Correlations between gene modules and disease phenotypes. **(E).** Lasso-penalized Cox regression analysis of the intersected genes. **(F, G).** The ROC curves of the DApred in training **(F)** and testing **(G)** sets. AUC, the area under the curve.


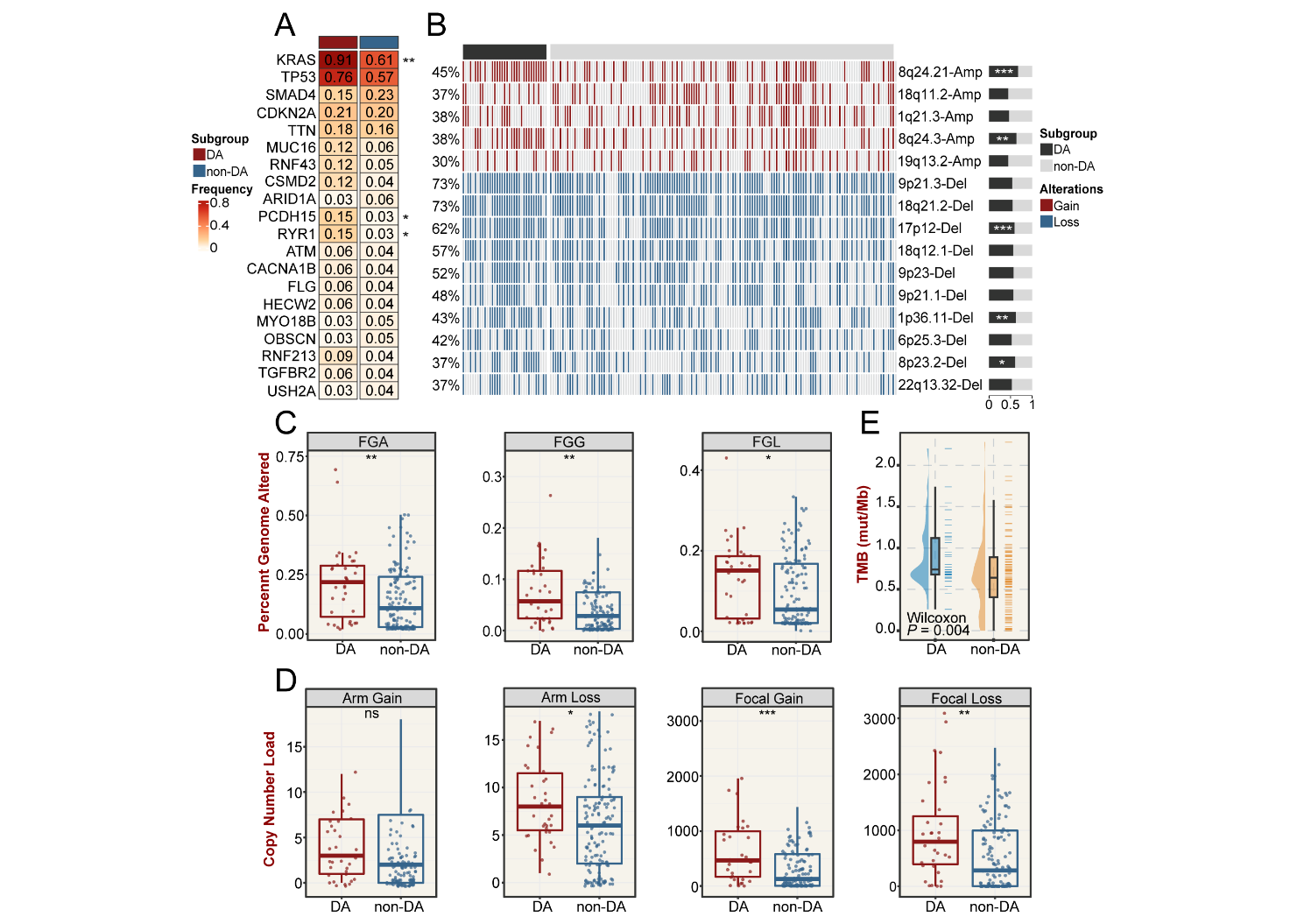


**Figure S3** The multi-omics landscape of the DA and non-DA subgroups. **(A).** Mutational frequency distribution of the FMGs between DA and non-DA subgroups. **(B).** The FAGs/FHGs in the DA and non-DA subgroups. **(C).** Distributions of FGA, FGG, and FGL between DA and non-DA. **(D).** Distributions of arm gain, arm loss, focal gain, and focal loss. **(E).** Distributions of TMB between DA and non-DA. DA, Double-Altered. non-DA, non-Double-Altered. AMP, amplification. HOMDEL, homozygous deletion. FGA, fraction of genome alteration. FGG, fraction of genome gained. FGL, fraction of genome lost. ^ns^*P*>0.05, ^*^*P* <0.05, ^**^*P* <0.01, ^***^*P* <0.001.


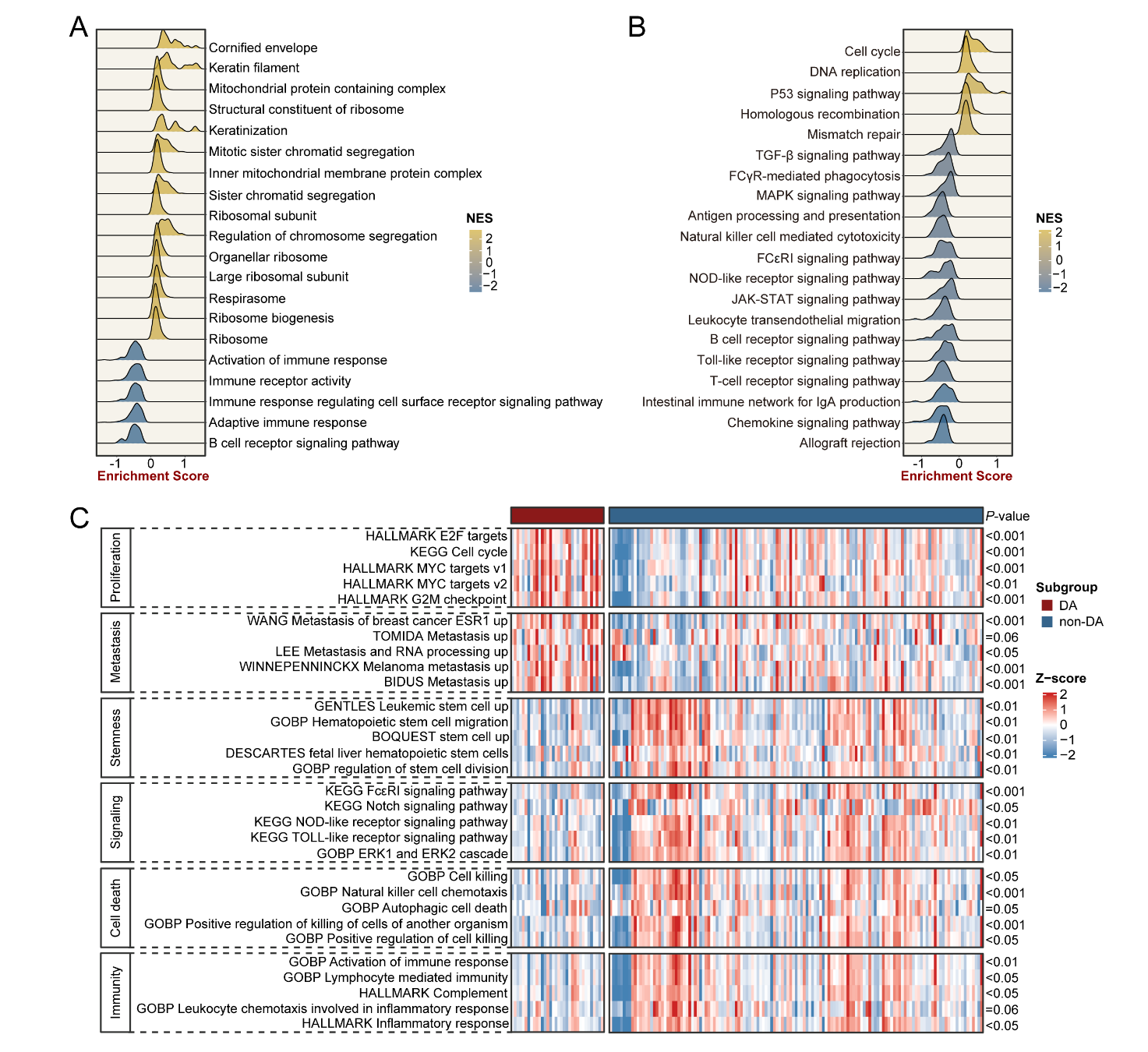


**Figure S4** Underlying biological pathways of the DA and non-DA subgroups. **(A, B).** GO **(A)** and KEGG **(B)** of gene set enrichment analysis (GSEA) according to the DA and non-DA subgroups. **(C).** Top 30 biological processes with significantly different enrichment between the DA and non-DA subgroups based on single-sample GSEA. GO, gene ontology. KEGG, Kyoto encyclopedia of genes and genomes. DA, Double-Altered. non-DA, non-Double-Altered.


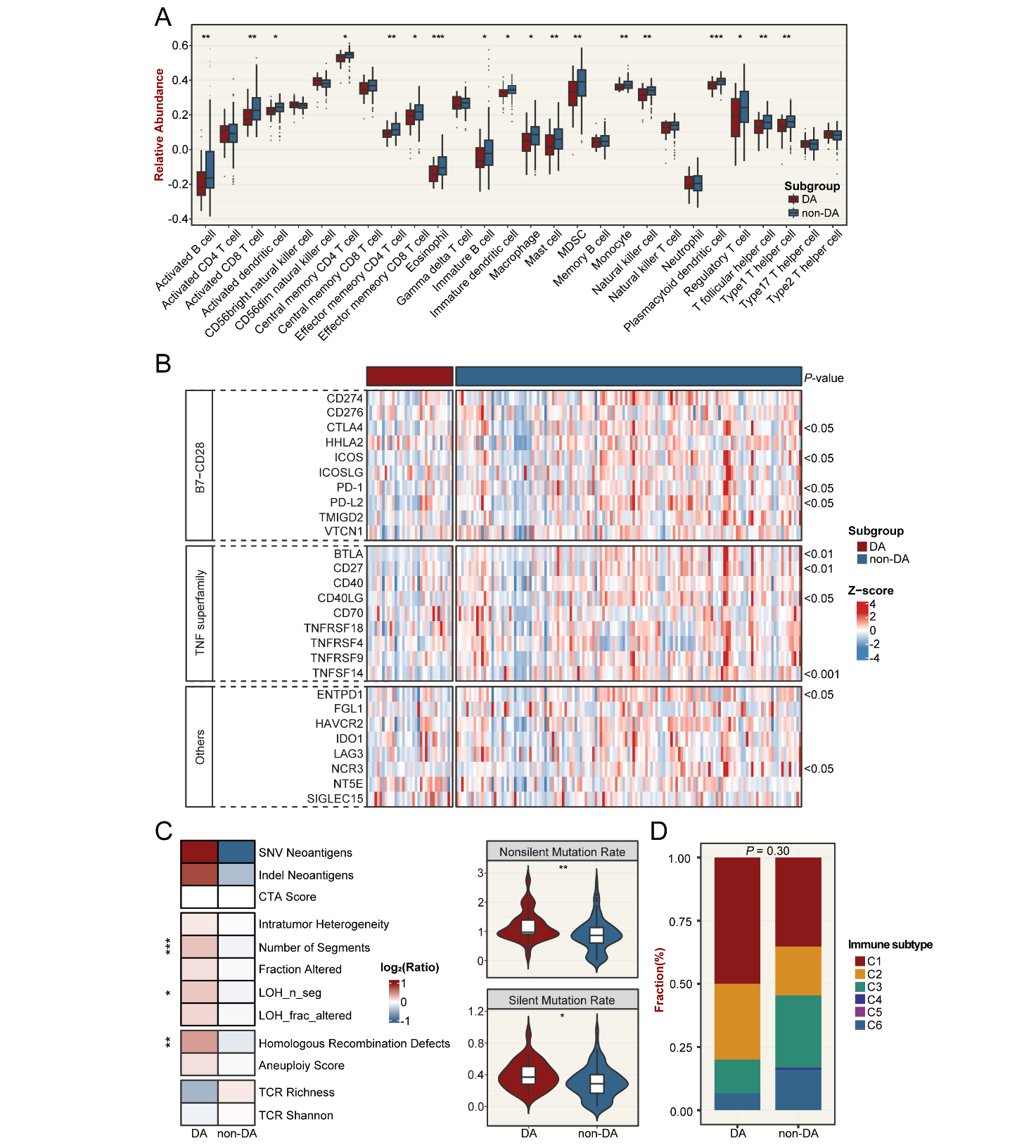


**Figure S5** The immune patterns of the DA and non-DA subgroups. **(A).** Distributions of 28 immune cells between the DA and non-DA subgroups. **(B).** The immune checkpoint profiles of the DA and non-DA subgroups. **(C).** The expression levels of 14 tumor antigenicity indicators of DA and non-DA. **(D).** Distribution of six immune subtypes in DA and non-DA. DA, Double-Altered. non-DA, non-Double-Altered. ^ns^*P*>0.05, ^*^*P* <0.05, ^**^*P* <0.01, ^***^*P* <0.001.
