# Supplementary material for: Co-alteration of Myc and RTK-RAS pathways defines a liver-metastatic propensity and immune-cold subgroup of pancreatic adenocarcinoma
Source: Genes Dis. 2023 Jun 29;11(3):100993. doi: 10.1016/j.gendis.2023.05.006 (PMC10806262; doi:10.1016/j.gendis.2023.05.006)
Supplement: Multimedia component 2 [file mmc2.docx]

| **Table S1. Details of baseline information in three independent cohorts.** | | | |
| --- | --- | --- | --- |
| **Accession** | **TCGA-PAAD** | **MSK-IMPACT** | **MSK-MET** |
| Number of Patients (%) | 179 (100.0) | 368 (100.0) | 1933 (100.0) |
| Event |  |  |  |
| Dead | 97 (54.2) | 102 (27.7) | 1174 (60.7) |
| Alive | 82 (45.8) | 266 (72.3) | 759 (39.3) |
| Age |  |  |  |
| >=65 | 96 (53.6) | -- | 1054 (54.5) |
| <65 | 83 (46.4) | -- | 879 (45.5) |
| Sex |  |  |  |
| Male | 97 (54.2) | 191 (51.9) | 1020 (52.8) |
| Female | 82 (45.8) | 177 (48.1) | 913 (47.2) |
| Stage |  |  |  |
| I | 20 (11.2) | -- | -- |
| II | 148 (82.7) | -- | -- |
| III | 4 (2.2) | -- | -- |
| IV | 5 (2.8) | -- | -- |
| Not available | 2 (1.1) | -- | -- |
| T stage |  |  |  |
| T1 | 6 (3.4) | -- | -- |
| T2 | 22 (12.3) | -- | -- |
| T3 | 145 (81.0) | -- | -- |
| T4 | 4 (2.2) | -- | -- |
| Not available | 2 (1.1) | -- | -- |
| N stage |  |  |  |
| N0 | 48 (26.8) | -- | -- |
| N1 | 126 (70.4) | -- | -- |
| Not available | 5 (2.8) | -- | -- |
| M stage |  |  |  |
| M0 | 82 (45.8) | -- | -- |
| M1 | 5 (2.8) | -- | -- |
| Not available | 92 (51.4) | -- | -- |
| Grade |  |  |  |
| G1 | 30 (16.8) | -- | -- |
| G2 | 94 (52.5) | -- | -- |
| G3 | 50 (27.9) | -- | -- |
| G4 | 2 (1.1) | -- | -- |
| Not available | 3 (1.7) | -- | -- |
| Sample Type |  |  |  |
| Metastasis | 0 (0.0) | 166 (45.1) | 795 (41.1) |
| Primary | 179 (100.0) | 202 (54.9) | 1138 (58.9) |
| Smoking |  |  |  |
| Yes | -- | 129 (35.1) | -- |
| No | -- | 164 (44.6) | -- |
| Not available | -- | 75 (20.4) | -- |
| Race |  |  |  |
| Asian | 11 (6.1) | -- | 111 (5.7) |
| Black or African American | 7 (3.9) | -- | 91 (4.7) |
| White | 156 (87.2) | -- | 1583 (81.9) |
| Not available | 5 (2.8) | -- | 148 (7.7) |
| Chronic pancreatitis |  |  |  |
| Yes | 13 (7.3) | -- | -- |
| No | 126 (70.4) | -- | -- |
| Not available | 40 (22.3) | -- | -- |
| Diabetes |  |  |  |
| Yes | 36 (20.1) | -- | -- |
| No | 109 (60.9) | -- | -- |
| Not available | 34 (19.0) | -- | -- |
| Alcohol |  |  |  |
| Yes | 100 (55.9) | -- | -- |
| No | 62 (34.6) | -- | -- |
| Not available | 17 (9.5) | -- | -- |
| MSI |  |  |  |
| Stable | -- | -- | 1645 (85.1) |
| Indeterminate | -- | -- | 33 (1.7) |
| Instable | -- | -- | 10 (0.5) |
| Not available | -- | -- | 245 (12.7) |

| **Table S2. Member genes of ten oncogenic signaling pathways.** | | |
| --- | --- | --- |
| **Pathway** | **Gene** | **OG/TSG** |
| Cell cycle | CDKN1A | TSG |
| Cell cycle | CDKN1B | TSG |
| Cell cycle | CDKN2A | TSG |
| Cell cycle | CDKN2B | TSG |
| Cell cycle | CDKN2C | TSG |
| Cell cycle | CCND1 | OG |
| Cell cycle | CCND2 | OG |
| Cell cycle | CCND3 | OG |
| Cell cycle | CCNE1 | OG |
| Cell cycle | CDK2 | OG |
| Cell cycle | CDK4 | OG |
| Cell cycle | CDK6 | OG |
| Cell cycle | RB1 | TSG |
| Cell cycle | E2F1 | OG |
| Cell cycle | E2F3 | OG |
| Hippo | STK4 | TSG |
| Hippo | STK3 | TSG |
| Hippo | SAV1 | TSG |
| Hippo | LATS1 | TSG |
| Hippo | LATS2 | TSG |
| Hippo | MOB1A | TSG |
| Hippo | MOB1B | TSG |
| Hippo | YAP1 | OG |
| Hippo | WWTR1 | OG |
| Hippo | TEAD1 | OG |
| Hippo | TEAD2 | OG |
| Hippo | TEAD3 | OG |
| Hippo | TEAD4 | OG |
| Hippo | PTPN14 | TSG |
| Hippo | NF2 | TSG |
| Hippo | WWC1 | TSG |
| Hippo | TAOK1 | TSG |
| Hippo | TAOK2 | TSG |
| Hippo | TAOK3 | TSG |
| Hippo | CRB1 | TSG |
| Hippo | CRB2 | TSG |
| Hippo | CRB3 | TSG |
| Hippo | LLGL1 | TSG |
| Hippo | LLGL2 | TSG |
| Hippo | HMCN1 | TSG |
| Hippo | SCRIB |  |
| Hippo | HIPK2 | OG |
| Hippo | FAT1 | TSG |
| Hippo | FAT2 | TSG |
| Hippo | FAT3 | TSG |
| Hippo | FAT4 | TSG |
| Hippo | DCHS1 | TSG |
| Hippo | DCHS2 | TSG |
| Hippo | CSNK1E | TSG |
| Hippo | CSNK1D | TSG |
| Hippo | AJUBA | OG |
| Hippo | LIMD1 | OG |
| Hippo | WTIP | OG |
| **Myc** | MAX | TSG |
| **Myc** | MGA | TSG |
| **Myc** | MLX | TSG |
| **Myc** | MLXIP | OG |
| **Myc** | MLXIPL | OG |
| **Myc** | MNT | TSG |
| **Myc** | MXD1 |  |
| **Myc** | MXD3 |  |
| **Myc** | MXD4 |  |
| **Myc** | MXI1 | TSG |
| **Myc** | MYC | OG |
| **Myc** | MYCL | OG |
| **Myc** | MYCN | OG |
| Notch | ARRDC1 | OG |
| Notch | CNTN6 | TSG |
| Notch | CREBBP | TSG |
| Notch | EP300 | TSG |
| Notch | HES1 | TSG |
| Notch | HES2 | TSG |
| Notch | HES3 | TSG |
| Notch | HES4 | TSG |
| Notch | HES5 | TSG |
| Notch | HEY1 | TSG |
| Notch | HEY2 | TSG |
| Notch | HEYL | TSG |
| Notch | KAT2B | TSG |
| Notch | KDM5A | OG |
| Notch | NOTCH1 | TSG |
| Notch | NOTCH2 | TSG |
| Notch | NOTCH3 | TSG |
| Notch | NOTCH4 | TSG |
| Notch | NOV | TSG |
| Notch | NRARP | OG |
| Notch | PSEN2 | TSG |
| Notch | LFNG |  |
| Notch | ITCH |  |
| Notch | NCSTN |  |
| Notch | SPEN | TSG |
| Notch | JAG1 |  |
| Notch | APH1A |  |
| Notch | FBXW7 | TSG |
| Notch | FHL1 |  |
| Notch | THBS2 |  |
| Notch | HDAC2 |  |
| Notch | MFAP2 |  |
| Notch | CUL1 | TSG |
| Notch | RFNG |  |
| Notch | NCOR1 | TSG |
| Notch | NCOR2 | TSG |
| Notch | MFAP5 |  |
| Notch | HDAC1 | OG |
| Notch | NUMB |  |
| Notch | JAG2 | TSG |
| Notch | MAML3 | TSG |
| Notch | MFNG |  |
| Notch | CIR1 |  |
| Notch | CNTN1 |  |
| Notch | MAML1 |  |
| Notch | MAML2 |  |
| Notch | NUMBL |  |
| Notch | PSEN1 |  |
| Notch | PSENEN | |
| Notch | RBPJ |  |
| Notch | RBPJL |  |
| Notch | RBX1 |  |
| Notch | SAP30 |  |
| Notch | SKP1 |  |
| Notch | SNW1 |  |
| Notch | CTBP1 |  |
| Notch | CTBP2 |  |
| Notch | ADAM10 | |
| Notch | APH1B |  |
| Notch | ADAM17 | |
| Notch | DLK1 |  |
| Notch | DLL1 |  |
| Notch | DLL3 |  |
| Notch | DLL4 |  |
| Notch | DNER | TSG |
| Notch | DTX1 |  |
| Notch | DTX2 |  |
| Notch | DTX3 |  |
| Notch | DTX3L |  |
| Notch | DTX4 |  |
| Notch | EGFL7 |  |
| Nrf2 | NFE2L2 | OG |
| Nrf2 | KEAP1 | TSG |
| Nrf2 | CUL3 | TSG |
| PI3K | EIF4EBP1 | OG |
| PI3K | AKT1 | OG |
| PI3K | AKT2 | OG |
| PI3K | AKT3 | OG |
| PI3K | AKT1S1 | OG |
| PI3K | DEPDC5 | |
| PI3K | DEPTOR | OG |
| PI3K | INPP4B | TSG |
| PI3K | MAPKAP1 | OG |
| PI3K | MLST8 | OG |
| PI3K | MTOR | OG |
| PI3K | NPRL2 | TSG |
| PI3K | NPRL3 | TSG |
| PI3K | PDK1 | OG |
| PI3K | PIK3CA | OG |
| PI3K | PIK3CB | OG |
| PI3K | PIK3R1 | TSG |
| PI3K | PIK3R2 | OG |
| PI3K | PIK3R3 | TSG |
| PI3K | PPP2R1A | TSG |
| PI3K | PTEN | TSG |
| PI3K | RHEB | OG |
| PI3K | RICTOR | OG |
| PI3K | RPTOR | OG |
| PI3K | RPS6 | OG |
| PI3K | RPS6KB1 | OG |
| PI3K | STK11 | TSG |
| PI3K | TSC1 | TSG |
| PI3K | TSC2 | TSG |
| TGFβ | TGFBR1 | TSG |
| TGFβ | TGFBR2 | TSG |
| TGFβ | ACVR2A | TSG |
| TGFβ | ACVR1B | TSG |
| TGFβ | SMAD2 | TSG |
| TGFβ | SMAD3 | TSG |
| TGFβ | SMAD4 | TSG |
| **RTK-RAS** | ABL1 | OG |
| **RTK-RAS** | EGFR | OG |
| **RTK-RAS** | ERBB2 | OG |
| **RTK-RAS** | ERBB3 | OG |
| **RTK-RAS** | ERBB4 | OG |
| **RTK-RAS** | PDGFRA | OG |
| **RTK-RAS** | PDGFRB | OG |
| **RTK-RAS** | MET | OG |
| **RTK-RAS** | FGFR1 | OG |
| **RTK-RAS** | FGFR2 | OG |
| **RTK-RAS** | FGFR3 | OG |
| **RTK-RAS** | FGFR4 | OG |
| **RTK-RAS** | FLT3 | OG |
| **RTK-RAS** | ALK | OG |
| **RTK-RAS** | RET | OG |
| **RTK-RAS** | ROS1 | OG |
| **RTK-RAS** | KIT | OG |
| **RTK-RAS** | IGF1R | OG |
| **RTK-RAS** | NTRK1 | OG |
| **RTK-RAS** | NTRK2 | OG |
| **RTK-RAS** | NTRK3 | OG |
| **RTK-RAS** | SOS1 | OG |
| **RTK-RAS** | GRB2 | OG |
| **RTK-RAS** | PTPN11 | OG |
| **RTK-RAS** | KRAS | OG |
| **RTK-RAS** | HRAS | OG |
| **RTK-RAS** | NRAS | OG |
| **RTK-RAS** | RIT1 | OG |
| **RTK-RAS** | ARAF | OG |
| **RTK-RAS** | BRAF | OG |
| **RTK-RAS** | RAF1 | OG |
| **RTK-RAS** | RAC1 | OG |
| **RTK-RAS** | MAP2K1 | OG |
| **RTK-RAS** | MAP2K2 | OG |
| **RTK-RAS** | MAPK1 | OG |
| **RTK-RAS** | NF1 | TSG |
| **RTK-RAS** | RASA1 | TSG |
| **RTK-RAS** | CBL | TSG |
| **RTK-RAS** | ERRFI1 | TSG |
| **RTK-RAS** | CBLB |  |
| **RTK-RAS** | CBLC |  |
| **RTK-RAS** | INSR |  |
| **RTK-RAS** | INSRR |  |
| **RTK-RAS** | IRS1 |  |
| **RTK-RAS** | SOS2 |  |
| **RTK-RAS** | SHC1 |  |
| **RTK-RAS** | SHC2 |  |
| **RTK-RAS** | SHC3 |  |
| **RTK-RAS** | SHC4 |  |
| **RTK-RAS** | RASGRP1 |  |
| **RTK-RAS** | RASGRP2 |  |
| **RTK-RAS** | RASGRP3 |  |
| **RTK-RAS** | RASGRP4 |  |
| **RTK-RAS** | RAPGEF1 |  |
| **RTK-RAS** | RAPGEF2 |  |
| **RTK-RAS** | RASGRF1 |  |
| **RTK-RAS** | RASGRF2 |  |
| **RTK-RAS** | FNTA |  |
| **RTK-RAS** | FNTB |  |
| **RTK-RAS** | RCE1 |  |
| **RTK-RAS** | ICMT |  |
| **RTK-RAS** | MRAS |  |
| **RTK-RAS** | PLXNB1 |  |
| **RTK-RAS** | MAPK3 |  |
| **RTK-RAS** | ARHGAP35 |  |
| **RTK-RAS** | RASA2 |  |
| **RTK-RAS** | RASA3 |  |
| **RTK-RAS** | RASAL1 |  |
| **RTK-RAS** | RASAL2 |  |
| **RTK-RAS** | RASAL3 |  |
| **RTK-RAS** | SPRED1 |  |
| **RTK-RAS** | SPRED2 |  |
| **RTK-RAS** | SPRED3 |  |
| **RTK-RAS** | DAB2IP |  |
| **RTK-RAS** | SHOC2 |  |
| **RTK-RAS** | PPP1CA |  |
| **RTK-RAS** | SCRIB |  |
| **RTK-RAS** | PIN1 |  |
| **RTK-RAS** | KSR1 |  |
| **RTK-RAS** | KSR2 |  |
| **RTK-RAS** | PEBP1 |  |
| **RTK-RAS** | ERF | TSG |
| **RTK-RAS** | PEA15 |  |
| **RTK-RAS** | JAK2 | OG |
| **RTK-RAS** | IRS2 | OG |
| p53 | TP53 | TSG |
| p53 | MDM2 | OG |
| p53 | MDM4 | OG |
| p53 | ATM | TSG |
| p53 | CHEK2 | TSG |
| p53 | RPS6KA3 | OG |
| Wnt | CHD8 |  |
| Wnt | LEF1 | OG |
| Wnt | LGR4 | OG |
| Wnt | LGR5 | OG |
| Wnt | LRP5 | OG |
| Wnt | LRP6 | OG |
| Wnt | LZTR1 |  |
| Wnt | NDP |  |
| Wnt | PORCN | OG |
| Wnt | RSPO1 | OG |
| Wnt | SFRP1 | TSG |
| Wnt | SFRP2 | TSG |
| Wnt | SFRP4 | TSG |
| Wnt | SFRP5 | TSG |
| Wnt | SOST | TSG |
| Wnt | TCF7L1 | TSG |
| Wnt | TLE1 | TSG |
| Wnt | TLE2 | TSG |
| Wnt | TLE3 | TSG |
| Wnt | TLE4 | TSG |
| Wnt | WIF1 | TSG |
| Wnt | ZNRF3 | TSG |
| Wnt | CTNNB1 | OG |
| Wnt | DVL1 | OG |
| Wnt | DVL2 | OG |
| Wnt | DVL3 | OG |
| Wnt | FRAT1 | OG |
| Wnt | FRAT2 | OG |
| Wnt | FZD1 | OG |
| Wnt | FZD10 | OG |
| Wnt | FZD2 | OG |
| Wnt | FZD3 | OG |
| Wnt | FZD4 | OG |
| Wnt | FZD5 | OG |
| Wnt | FZD6 | OG |
| Wnt | FZD7 | OG |
| Wnt | FZD8 | OG |
| Wnt | FZD9 | OG |
| Wnt | WNT1 | OG |
| Wnt | WNT10A | OG |
| Wnt | WNT10B | OG |
| Wnt | WNT11 | OG |
| Wnt | WNT16 | OG |
| Wnt | WNT2 | OG |
| Wnt | WNT3A | OG |
| Wnt | WNT4 | OG |
| Wnt | WNT5A | OG |
| Wnt | WNT5B | OG |
| Wnt | WNT6 | OG |
| Wnt | WNT7A | OG |
| Wnt | WNT7B | OG |
| Wnt | WNT8A | OG |
| Wnt | WNT8B | OG |
| Wnt | WNT9A | OG |
| Wnt | WNT9B | OG |
| Wnt | AMER1 | TSG |
| Wnt | APC | TSG |
| Wnt | AXIN1 | TSG |
| Wnt | AXIN2 | TSG |
| Wnt | DKK1 | TSG |
| Wnt | DKK2 | TSG |
| Wnt | DKK3 | TSG |
| Wnt | DKK4 | TSG |
| Wnt | GSK3B | TSG |
| Wnt | RNF43 | TSG |
| Wnt | TCF7 | TSG |
| Wnt | TCF7L2 | TSG |
| Wnt | CHD4 |  |
|  |  |  |
| OG: Oncogene | |  |
| TSG: Tumor suppressor | | |

| **Table S3. Co-occurring and mutually exclusive pathways identified by SELECT.**   \| **Pathyway pair** \| **wMI_p.value** \| **ASC_good** \| **direction** \| **Surv_p.value** \| \| --- \| --- \| --- \| --- \| --- \| \| Cell cycle & Hippo \| <0.001 \| TRUE \| co-occurrence \| 0.2627631 \| \| Cell cycle & RTK-RAS \| <0.001 \| TRUE \| co-occurrence \| 0.0367328 \| \| Hippo & Myc \| <0.001 \| TRUE \| co-occurrence \| 0.0056506 \| \| Hippo & Notch \| <0.001 \| FALSE \| co-occurrence \| 0.6316506 \| \| Hippo & PI3K \| <0.001 \| FALSE \| co-occurrence \| 0.4574943 \| \| Hippo & Wnt \| <0.001 \| TRUE \| co-occurrence \| 0.6597554 \| \| Myc & Notch \| <0.001 \| TRUE \| co-occurrence \| 0.1027202 \| \| Myc & PI3K \| <0.001 \| TRUE \| co-occurrence \| 0.0132654 \| \| Notch & PI3K \| <0.001 \| TRUE \| co-occurrence \| 0.0444104 \| \| Notch & Wnt \| <0.001 \| TRUE \| co-occurrence \| 0.6915246 \| \| PI3K & Wnt \| <0.001 \| TRUE \| co-occurrence \| 0.1811039 \| \| Cell cycle & Wnt \| 0.001 \| TRUE \| co-occurrence \| 0.1261287 \| \| Hippo & RTK-RAS \| 0.002 \| FALSE \| co-occurrence \| 0.17596 \| \| Myc & Nrf2 \| 0.005 \| TRUE \| co-occurrence \| 0.0140356 \| \| **Myc & RTK-RAS** \| **0.006** \| **TRUE** \| **co-occurrence** \| **0.0032581** \| \| Cell cycle & Notch \| 0.007 \| FALSE \| co-occurrence \| 0.2681573 \| \| Myc & Wnt \| 0.009 \| FALSE \| co-occurrence \| 0.0230732 \| \| Cell cycle & p53 \| 0.011 \| FALSE \| co-occurrence \| 0.0169765 \| \| Hippo & p53 \| 0.02 \| FALSE \| co-occurrence \| 0.2490429 \| \| Cell cycle & PI3K \| 0.021 \| FALSE \| co-occurrence \| 0.0932838 \| \| Notch & p53 \| 0.026 \| FALSE \| co-occurrence \| 0.3416324 \| \| Notch & RTK-RAS \| 0.046 \| FALSE \| co-occurrence \| 0.1972379 \| \| Nrf2 & TGFβ \| 0.049 \| FALSE \| co-occurrence \| 0.3188695 \| \| RTK-RAS & TGFβ \| 0.052 \| TRUE \| co-occurrence \| 0.0443283 \| \| PI3K & TGFβ \| 0.082 \| FALSE \| mutual exclusivity \| 0.2082221 \| \| Cell cycle & TGFβ \| 0.091 \| TRUE \| co-occurrence \| 0.0973672 \| \| Nrf2 & Wnt \| 0.126 \| FALSE \| co-occurrence \| 0.5775165 \| \| RTK-RAS & p53 \| 0.137 \| TRUE \| co-occurrence \| 0.0618525 \| \| RTK-RAS & Wnt \| 0.206 \| FALSE \| co-occurrence \| 0.1181562 \| \| PI3K & RTK-RAS \| 0.212 \| FALSE \| co-occurrence \| 0.1141139 \| \| PI3K & p53 \| 0.219 \| FALSE \| co-occurrence \| 0.1991345 \| \| Hippo & Nrf2 \| 0.257 \| FALSE \| co-occurrence \| 0.7974154 \| \| Myc & p53 \| 0.281 \| FALSE \| co-occurrence \| 0.0099577 \| \| Cell cycle & Myc \| 0.427 \| FALSE \| co-occurrence \| 0.0052538 \| \| TGFβ & Wnt \| 0.432 \| FALSE \| co-occurrence \| 0.3030573 \| \| Notch & Nrf2 \| 0.441 \| FALSE \| co-occurrence \| 0.6614078 \| \| Nrf2 & RTK-RAS \| 0.46 \| FALSE \| co-occurrence \| 0.0466962 \| \| p53 & Wnt \| 0.491 \| FALSE \| co-occurrence \| 0.2195907 \| \| Hippo & TGFβ \| 0.526 \| FALSE \| co-occurrence \| 0.4243321 \| \| Nrf2 & p53 \| 0.668 \| TRUE \| co-occurrence \| 0.2002087 \| \| Cell cycle & Nrf2 \| 0.691 \| FALSE \| mutual exclusivity \| 0.0760219 \| \| Nrf2 & PI3K \| 0.692 \| FALSE \| co-occurrence \| 0.4801177 \| \| Notch & TGFβ \| 0.77 \| FALSE \| mutual exclusivity \| 0.2937035 \| \| Myc & TGFβ \| 0.856 \| FALSE \| co-occurrence \| 0.0203938 \| \| p53 & TGFβ \| 0.883 \| FALSE \| mutual exclusivity \| 0.0605795 \| | |
| --- | --- | --- | --- | --- | --- | --- | --- | --- | --- | --- | --- | --- | --- | --- | --- | --- | --- | --- | --- | --- | --- | --- | --- | --- | --- | --- | --- | --- | --- | --- | --- | --- | --- | --- | --- | --- | --- | --- | --- | --- | --- | --- | --- | --- | --- | --- | --- | --- | --- | --- | --- | --- | --- | --- | --- | --- | --- | --- | --- | --- | --- | --- | --- | --- | --- | --- | --- | --- | --- | --- | --- | --- | --- | --- | --- | --- | --- | --- | --- | --- | --- | --- | --- | --- | --- | --- | --- | --- | --- | --- | --- | --- | --- | --- | --- | --- | --- | --- | --- | --- | --- | --- | --- | --- | --- | --- | --- | --- | --- | --- | --- | --- | --- | --- | --- | --- | --- | --- | --- | --- | --- | --- | --- | --- | --- | --- | --- | --- | --- | --- | --- | --- | --- | --- | --- | --- | --- | --- | --- | --- | --- | --- | --- | --- | --- | --- | --- | --- | --- | --- | --- | --- | --- | --- | --- | --- | --- | --- | --- | --- | --- | --- | --- | --- | --- | --- | --- | --- | --- | --- | --- | --- | --- | --- | --- | --- | --- | --- | --- | --- | --- | --- | --- | --- | --- | --- | --- | --- | --- | --- | --- | --- | --- | --- | --- | --- | --- | --- | --- | --- | --- | --- | --- | --- | --- | --- | --- | --- | --- | --- | --- | --- | --- | --- | --- | --- | --- | --- | --- | --- | --- | --- | --- | --- | --- | --- | --- | --- | --- | --- | --- |
| **Table S4.** **Comparison of metastasis rates at 20 sites of DA and non-DA in the MSK-MET cohort.** |  |

| **Metastatic sites** | **P-Value** |
| --- | --- |
| Adrenal Gland | 0.4742 |
| Biliary Tract | 0.3453 |
| Bladder/Uterus | 1 |
| Bone | 0.875 |
| Bowel | 0.471 |
| Breast | 1 |
| CNS Brain | 0.2946 |
| Distant Lymph Node | 0.5847 |
| Female Genital | 1 |
| Head and Neck | 1 |
| **Peritoneum** | 0.1691 |
| Kidney | 1 |
| **Liver** | **0.04313** |
| Lung | 0.8562 |
| Male Genital | 1 |
| Mediastinum | 0.116 |
| Ovary | 0.2051 |
| Pleura | 0.4838 |
| PNS | 0.5989 |
| Skin | 1 |

| **Table S5. Genes from blue and purple modules with AUC >0.7 for diagnosis of DA.** | |
| --- | --- |
| **Gene** | **AUC** |
| ABCA9 | 0.74195 |
| ADAM32 | 0.71146 |
| ADH1B | 0.70706 |
| AHCY | 0.70781 |
| ALDOA | 0.70288 |
| B3GNT3 | 0.72714 |
| C10orf105 | 0.7018 |
| C7 | 0.70331 |
| CACNB4 | 0.70932 |
| CCIN | 0.7179 |
| CCNB1 | 0.7018 |
| CCT8 | 0.7179 |
| CD300LB | 0.70416 |
| CDCA3 | 0.70803 |
| CENPA | 0.70073 |
| CFP | 0.72821 |
| CHCHD4 | 0.7164 |
| CKS1B | 0.70245 |
| CLEC10A | 0.70052 |
| CLEC9A | 0.70352 |
| CLIC1 | 0.7134 |
| CLNK | 0.71833 |
| CMBL | 0.70288 |
| CTC1 | 0.70094 |
| CTNS | 0.72585 |
| CXCL12 | 0.73229 |
| CYC1 | 0.70073 |
| DNASE1L3 | 0.70867 |
| EBF3 | 0.7237 |
| EFNA3 | 0.70588 |
| ELAPOR2 | 0.70696 |
| FAM162A | 0.70889 |
| FARSB | 0.7207 |
| FCRL6 | 0.7076 |
| FKBP4 | 0.7179 |
| GAPDH | 0.70202 |
| GCNA | 0.71254 |
| GFUS | 0.70867 |
| GPR182 | 0.70245 |
| HJURP | 0.71812 |
| HSPB6 | 0.70824 |
| HSPD1 | 0.71189 |
| HSPE1 | 0.73486 |
| IGSF10 | 0.71404 |
| KCNJ5 | 0.70266 |
| KLRC4-KLRK1 | 0.71146 |
| KSR1 | 0.73358 |
| LIMD2 | 0.71254 |
| LRATD2 | 0.73766 |
| LRRC1 | 0.7252 |
| LRRC55 | 0.71232 |
| LSM4 | 0.71769 |
| LY6G6C | 0.71093 |
| MAD2L1 | 0.71103 |
| MAL2 | 0.72413 |
| MIS18A | 0.71898 |
| MKNK1 | 0.71018 |
| MPZ | 0.71297 |
| MRGBP | 0.70481 |
| MRPL13 | 0.70653 |
| MRPS35 | 0.75676 |
| MSC | 0.71662 |
| NDRG1 | 0.74925 |
| NDUFB9 | 0.74023 |
| NPY5R | 0.70223 |
| NR2C2AP | 0.70288 |
| OMG | 0.7149 |
| PDE7B | 0.72907 |
| PLD2 | 0.70202 |
| PLIN1 | 0.70416 |
| PLPP3 | 0.72585 |
| POC1A | 0.70459 |
| PRELID3B | 0.70416 |
| PRXL2A | 0.70137 |
| PTGFR | 0.70481 |
| PTK6 | 0.70545 |
| PTTG1 | 0.70588 |
| RGL4 | 0.72263 |
| RNASEH2A | 0.70052 |
| SCARF1 | 0.71511 |
| SEPTIN1 | 0.72606 |
| SERPINB5 | 0.73497 |
| SGO1 | 0.70631 |
| SLC43A2 | 0.74453 |
| SPAG7 | 0.70781 |
| STYXL1 | 0.7368 |
| TDRD10 | 0.71297 |
| TDRD6 | 0.70803 |
| TGFBR3L | 0.70889 |
| TNNT1 | 0.72177 |
| TPX2 | 0.70374 |
| TREML1 | 0.70331 |
| TUBA4A | 0.73916 |
| VDAC1 | 0.71468 |
| VDAC2 | 0.70846 |
| ZNF488 | 0.70717 |
| ZNF57 | 0.73315 |

| **Table S6. 14 genes were identified based on the selected optimal lambda of 0.020 (DApred).** |
| --- |
| **Gene** |
| TNNT1 |
| ZNF488 |
| MAL2 |
| NDRG1 |
| LRATD2 |
| ALDOA |
| MAD2L1 |
| PTK6 |
| EFNA3 |
| ELAPOR2 |
| LIMD2 |
| LRRC55 |
| PLPP3 |
| PDE7B |

| **Table S7. The details of indicators for the assessment of immunogenicity and antigen presentation capacity.** | | |  |
| --- | --- | --- | --- |
| **Indicator** | **Details** | **Reference(s) [PMID]** | |
| Nonsilent Mutation Rate | - | https://pubmed.ncbi.nlm.nih.gov/29628290 | |
| Silent Mutation Rate | - | https://pubmed.ncbi.nlm.nih.gov/29628290 | |
| Wound Healing | The values of Wound Healing reflect the characteristics of "Immune Subtype" C1 | https://pubmed.ncbi.nlm.nih.gov/29628290 | |
| Proliferation | - | https://pubmed.ncbi.nlm.nih.gov/29628290 | |
| Immune Subtype | Using data compiled by TCGA, an extensive immunohistochemical analysis was performed on more than 10000 tumors including 33 different cancer types. In all cancer types, six immune subtypes were identified: Wound Healing, IFN-γ Dominant, Inflammatory, Lymphocyte Depleted, Immunologically Quiet, and TGF-β Dominant. | https://pubmed.ncbi.nlm.nih.gov/29628290 | |
| SNV Neoantigens | Single nucleotide variation (SNV) neoantigens were identified through NetMHCpan v3.0,based on HLA types obtained from RNA-seq using OptiType (version 1.2) | https://pubmed.ncbi.nlm.nih.gov/29628290; https://pubmed.ncbi.nlm.nih.gov/27029192; https://pubmed.ncbi.nlm.nih.gov/25143287 | |
| Indel Neoantigens | Insertion-deletion (indel) neoantigens were identified through NetMHCpan v3.0,based on HLA types obtained from RNA-seq using OptiType (version 1.2) | https://pubmed.ncbi.nlm.nih.gov/29628290; https://pubmed.ncbi.nlm.nih.gov/27029192; https://pubmed.ncbi.nlm.nih.gov/25143287 | |
| CTA score | Cancer/testis-antigen (CTA) | https://pubmed.ncbi.nlm.nih.gov/29628290 | |
| Intratumor Heterogeneity | Intratumor genetic heterogeneity (ITH) is a feature of tumors that refers to the repertoire of co-existing genetically distinct subclonal populations. | https://pubmed.ncbi.nlm.nih.gov/29628290; https://pubmed.ncbi.nlm.nih.gov/26840267 | |
| Number of Segs | Number of copy number variant segments | https://pubmed.ncbi.nlm.nih.gov/29628290 | |
| Fraction Altered | Fraction of genome alterations | https://pubmed.ncbi.nlm.nih.gov/29628290 | |
| Homologous Recombination Defects | Homologous recombination defects (HRD) score was determined by three separate DNA-based measures of genomic instability: large (> 15 Mb) non-arm-level regions with loss of heterozygosity (LOH), telomeric allelic imbalance (TAI), and large-scale state transitions (LST) with breaks between adjacent segments of > 10 Mb | https://pubmed.ncbi.nlm.nih.gov/29628290 | |
| Aneuploidy score | Aneuploidy scores (AS) were the sum of amplified or deleted (collectively "altered") chromosome arms | https://pubmed.ncbi.nlm.nih.gov/29628290; https://pubmed.ncbi.nlm.nih.gov/29622463 | |
| TCR Richness | TCR diversity (Richness) scores were identified using MiTCR v1.0.3, with previously described parameters | https://pubmed.ncbi.nlm.nih.gov/29628290; https://pubmed.ncbi.nlm.nih.gov/23892897; https://pubmed.ncbi.nlm.nih.gov/25196070 | |
| TCR Shannon | TCR diversity (Shannon Entropy) scores were identified using MiTCR v1.0.3, with previously described parameters | https://pubmed.ncbi.nlm.nih.gov/29628290; https://pubmed.ncbi.nlm.nih.gov/23892897; https://pubmed.ncbi.nlm.nih.gov/25196070 | |
| Number of Segs with LOH | Number of Segs with loss of heterozygosity (LOH) | https://pubmed.ncbi.nlm.nih.gov/29622463 | |
| Fraction of Segs with LOH | Fraction of Segs with loss of heterozygosity (LOH) | https://pubmed.ncbi.nlm.nih.gov/29622463 | |
