# Supplementary material for: Co-alteration of Myc and RTK-RAS pathways defines a liver-metastatic propensity and immune-cold subgroup of pancreatic adenocarcinoma
Source: Genes Dis. 2023 Jun 29;11(3):100993. doi: 10.1016/j.gendis.2023.05.006 (PMC10806262; doi:10.1016/j.gendis.2023.05.006)
Supplement: Multimedia component 3 [file mmc3.docx]

**Materials and Methods**

**Data collection and processing**

The cBioPortal for Cancer Genomics (www.cbioportal.org) contains plentiful multi-omics data on pancreatic adenocarcinoma (PAAD), which was utilized to screen the eligible cohort that met the following conditions: (i) containing somatic mutation, copy number variation (CNV, GISITC 2.0), and gene fusion or rearrangement data; (ii) containing clinical data with survival information; (iii) samples without undergone neoadjuvant chemotherapy; (iv) the number of samples exceeding 100. In total, three independent cohorts were obtained, including TCGA-PAAD (n =179), MSK-IMPACT (n =368), and MSK-MET (n =1933). The detailed baseline of all cohorts was available in Table S1. In addition, for the TCGA-PAAD cohort, RNA-Seq data (FPKM) downloaded from the UCSC Xena (https://xenabrowser.net/datapages/) was transformed into transcripts per kilobase million (TPM), and further log2 (TPM+1) format.

**SELECT Algorithm and Defining Oncogenic Pathway Alterations**

The R-based SELECT algorithm (version 1.0) was publicly available and downloaded from the website (ciriellolab.org/select/select.html). Using this tool, we aimed to explore the synergic and/or antagonistic alterations of ten oncogenic pathways in TCGA-PAAD samples, including cell cycle, Hippo, Myc, Notch, Nrf2, PI-3-Kinase/Akt, RTK-RAS, TGFβ signaling, p53, and β-catenin/WNT.

Before that, modeling after the previous approach^1^, the signaling pathway was considered “altered” in PAAD if any of the following occurred in its pathway member genes (Table S2): (i) non-synonymous mutation; (ii) copy number amplification (GISTIC calls >=2) or deletion (GISTIC calls <=−2); and (iii) gene fusions or rearrangements.

The overview of the SELECT workflow was similar to Mina et al.^2^: After entering a binary matrix containing the altered status of 179 samples in 10 canonical pathways (each row represents a sample and each column a pathway. 0 in the matrix represents unaltered and 1 represents altered), SELECT first paired pathways against each other and assigned each pathway pair an observed wMI value to assess the extent to which one altered state influenced another. Secondly, to determine whether the conclusion was due to chance, observed wMI values were compared with the expected wMI values calculated from the randomized binary matrix (which was randomly repeated 1000 times). Define the percentage of the expected wMI value greater than or equal to the observed wMI value as the ‘wMI_p.value’ for the pathway pair. Finally, the Average Sum Correction (ASC) was applied to calculate the ASC score of a pathway pair and estimate whether there was a true and direct evolutionary dependency between two oncogenic pathways. The ‘ASC_good’ indicated whether the ASC score exceeded the threshold established by SELECT. The ‘direction’ showed the biological evolutionary pattern of PAAD oncogenic pathway pairs.

PAAD samples with both pathways altered were termed “Double-Altered” (DA), those with neither pathway altered were termed “Double-Unaltered” (DU), and the others were termed “Single-Altered” (SA). The ‘Surv_p.value’ of each path pair was obtained by the survival analysis of three subgroups (DA, SA, and DU). Whenever DA, SA, DU, and non-DA(SA/DU) were mentioned in the text, it always refers to the subgroup of the Myc & RTK-RAS pathway pair.

**Model Construction of the Predictor**

Weighted Gene Co-expression Network Analysis (WGCNA) was implemented via the WGCNA package to explore markers with clinicobiological features^3^. The TCGA-PAAD outliers whose dendrogram height exceeded 140 were removed via the “hclust” function. The optimal β was picked to satisfy the scale-free distribution. Based on Pearson’s correlation between extracted gene pairs, the adjacency matrix was constructed as a topological overlap matrix (TOM) and a corresponding dissimilarity matrix (1-TOM). The cutreeDynamic function was utilized to incorporate similar co-expression modules. Finally, the hierarchical clustering method was employed to categorize genes into different modules. The module Eigengene (ME) was calculated, which represented the gene expression profile of each module.

The modules highly related to the subgroup were the key modules and then the area under the curve (AUC) values of genes therein were calculated. Genes with an AUC value greater than 0.7 were reserved for the LASSO logistic regression analysis using the *glmnet* package. Subsequently, the TCGA-PAAD samples were randomized to training and test sets at a ratio of 7:3 by the *caret* package. The generation of the receiver operating characteristic (ROC) curve and AUC calculation were realized by the *pROC* package.

**Functional enrichment analysis**

Gene set enrichment analysis (GSEA) and gene set variation analysis (GSVA) were carried out to elaborate on the gene sets associated with the DA subgroup in the TCGA-PAAD cohort. The gene sets were obtained from MSigDB resource (<http://software.broadinstitute.org/gsea/msigdb>), including go.v7.5.1 and kegg.v7.5.1. All genes were sorted by descending order (log2-transformation fold change), which was calculated between the DA and non-DA subgroups using the *limma* package. Then, the ranked gene list was used to explore enriched pathways by the *clusterProfiler* package and only terms with adjusted *P* value <0.05 were retained. Afterward, 30 GMT files from the MSigDB database were selected to perform GSVA via the *GSVA* package.

**Multi-omics Landscape of DA and Calculation of TMB**

To decipher the multi-omics landscape of DA, the mutation and CNV data in the TCGA-PAAD cohort were integratively analyzed. The previously published studies defined the frequently mutated genes (FMGs) as genes with the top-20 mutational frequency^4,5^. After running GISTIC 2.0 module on the GenePattern platform (https://cloud.genepattern.org/gp), 15 chromosome segments with the highest broad-level CNA frequency were displayed. Based on our proposed pipeline^5^, to quantify genomic alterations in DA, we calculated the ratio of total CNV/gain/loss bases to all bases, referred to as the fraction of genome altered (FGA), the fraction of genome gained (FGG), and the fraction of genome lost (FGL), respectively. Additionally, the burdens with CNV were quantified at the arm and focal levels based on the recurrently changed regions originating from the GISTIC 2.0 pipeline. Tumor mutational burden (TMB) was defined as the number of non-synonymous somatic mutations per million bases^6^. The TMB of each patient was calculated using the “tmb” function in the maftools package^7^.

**Tumor Immune Microenvironment Profiles and Acquisition of Immune-related Indicators**

To quantify the level of immune cell infiltration in TCGA-PAAD samples, gene sets on 28 immune cells in innate immunity^8^ were gathered to perform single-sample GSEA (ssGSEA) via the *GSVA* package. To assess differences in overall genomic instability and tumor antigenicity between the two subgroups, multiple measures from Thorsson V et al. were recruited (Table S7), including SNV neoantigens, non-silent mutation rate, silent mutation rate, and so on^9-11^.

**Statistical Analysis**

All data processing, statistical analysis, and graphical visualization were conducted in R (version 4.2.1). The Kaplan–Meier method and the log-rank test were utilized to assess the different survival between the two subgroups. The difference between the two subgroups was tested by performing the Wilcoxon rank-sum test or Student’s t-test for continuous variables, and the chi-squared test or Fisher’s exact test for categorical variables.

**References:**

1. Sanchez-Vega F, Mina M, Armenia J, et al. Oncogenic Signaling Pathways in The Cancer Genome Atlas. *Cell.* 2018; <https://doi.org/10.1016/j.cell.2018.03.035>.

2. Mina M, Raynaud F, Tavernari D, et al. Conditional Selection of Genomic Alterations Dictates Cancer Evolution and Oncogenic Dependencies. *Cancer Cell.* 2017; <https://doi.org/10.1016/j.ccell.2017.06.010>.

3. Langfelder P, Horvath S. WGCNA: an R package for weighted correlation network analysis. *BMC Bioinformatics.* 2008; <https://doi.org/10.1186/1471-2105-9-559>.

4. Liu Z, Wang L, Guo C, et al. TTN/OBSCN 'Double-Hit' predicts favourable prognosis, 'immune-hot' subtype and potentially better immunotherapeutic efficacy in colorectal cancer. *J Cell Mol Med.* 2021; <https://doi.org/10.1111/jcmm.16393>.

5. Liu Z, Xu H, Weng S, Ren Y, Han X. Stemness Refines the Classification of Colorectal Cancer With Stratified Prognosis, Multi-Omics Landscape, Potential Mechanisms, and Treatment Options. *Front Immunol.* 2022; <https://doi.org/10.3389/fimmu.2022.828330>.

6. Attalla K, DiNatale RG, Rappold PM, et al. Prevalence and Landscape of Actionable Genomic Alterations in Renal Cell Carcinoma. *Clin Cancer Res.* 2021; <https://doi.org/10.1158/1078-0432.CCR-20-4058>.

7. Mayakonda A, Lin DC, Assenov Y, Plass C, Koeffler HP. Maftools: efficient and comprehensive analysis of somatic variants in cancer. *Genome Res.* 2018; <https://doi.org/10.1101/gr.239244.118>.

8. Charoentong P, Finotello F, Angelova M, et al. Pan-cancer Immunogenomic Analyses Reveal Genotype-Immunophenotype Relationships and Predictors of Response to Checkpoint Blockade. *Cell Rep.* 2017; <https://doi.org/10.1016/j.celrep.2016.12.019>.

9. Wang S, He Z, Wang X, Li H, Liu XS. Antigen presentation and tumor immunogenicity in cancer immunotherapy response prediction. *Elife.* 2019; <https://doi.org/10.7554/eLife.49020>.

10. Thorsson V, Gibbs DL, Brown SD, et al. The Immune Landscape of Cancer. *Immunity.* 2018; <https://doi.org/10.1016/j.immuni.2018.03.023>.

11. Taylor AM, Shih J, Ha G, et al. Genomic and Functional Approaches to Understanding Cancer Aneuploidy. *Cancer Cell.* 2018; <https://doi.org/10.1016/j.ccell.2018.03.007>.
